# LncRNA DPP10-AS1 promotes malignant processes through epigenetically activating its cognate gene DPP10 and predicts poor prognosis in lung cancer patients

**DOI:** 10.20892/j.issn.2095-3941.2020.0136

**Published:** 2021-08-15

**Authors:** Haihua Tian, Jinchang Pan, Shuai Fang, Chengwei Zhou, Hui Tian, Jinxian He, Weiyu Shen, Xiaodan Meng, Xiaofeng Jin, Zhaohui Gong

**Affiliations:** 1 Department of Biochemistry and Molecular Biology, Ningbo University School of Medicine, Ningbo 315211, China; 2 Zhejiang Province Key Laboratory of Pathophysiology, Ningbo University School of Medicine, Ningbo 315211, China; 3 Department of Thoracic Surgery, The Affiliated Hospital of Ningbo University School of Medicine, Ningbo 315020, China; 4 Department of Thoracic Surgery, The Affiliated Lihuili Hospital of Ningbo University, Ningbo 315048, China

**Keywords:** Antisense long noncoding RNA, DPP10-AS1, hypomethylation, malignant process, lung cancer

## Abstract

**Objective:**

The purpose of this study was to explore the function and gene expression regulation of the newly identified lncRNA DPP10-AS1 in lung cancer, and its potential value as a prognostic biomarker.

**Methods:**

qRT-PCR and Western blot were conducted to detect the expression of DDP10-AS1 and DPP10 in lung cancer cell lines and tissues. The effects of DDP10-AS1 on DPP10 expression, cell growth, invasion, apoptosis, and *in vivo* tumor growth were investigated in lung cancer cells by Western blot, rescue experiments, colony formation, flow cytometry, and xenograft animal experiments.

**Results:**

The novel antisense lncRNA DPP10-AS1 was found to be highly expressed in cancer tissues (*P* < 0.0001), and its upregulation predicted poor prognosis in patients with lung cancer (*P* = 0.0025). Notably, DPP10-AS1 promoted lung cancer cell growth, colony formation, and cell cycle progression, and repressed apoptosis in lung cancer cells by upregulating DPP10 expression. Additionally, DPP10-AS1 facilitated lung tumor growth *via* upregulation of DPP10 protein in a xenograft mouse model. Importantly, DPP10-AS1 positively regulated *DPP10* gene expression, and both were coordinately upregulated in lung cancer tissues. Mechanically, DPP10-AS1 was found to associate with *DPP10* mRNA but did not enhance *DPP10* mRNA stability. Hypomethylation of DPP10-AS1 and *DPP10* contributed to their coordinate upregulation in lung cancer.

**Conclusions:**

These findings indicated that the upregulation of the antisense lncRNA DPP10-AS1 promotes lung cancer malignant processes and facilitates tumorigenesis by epigenetically regulating its cognate sense gene *DPP10*. DPP10-AS1 may serve as a candidate prognostic biomarker and a potential therapeutic target in lung cancer.

## Introduction

Long noncoding RNAs (lncRNAs) are a class of non-coding RNAs comprising > 200 nucleotides with limited protein-coding ability^1,2^. Although these lncRNAs were once regarded as transcriptional noise^3^, studies have since demonstrated that lncRNAs participate in various biological processes, including epigenetic control, the regulation of gene expression, RNA maturation (including splicing and editing), and the maintenance of chromatin structure^4–6^. LncRNAs are key regulators in the development and progression of cancers through pathophysiological activities such as cell growth, apoptosis, invasion, and metastasis^7,8^. Previous studies have demonstrated that lncRNAs such as HOTAIR (Hox transcript antisense intergenic RNA) and MALAT1 (metastasis-associated lung adenocarcinoma transcript 1) are upregulated in lung cancer; are associated with enhanced proliferation and metastasis; and predict poor prognosis^9,10^. Another lncRNA, MIR22HG, stabilizes the YBX1 protein and predicts poor survival in patients with lung cancer^11^. Antisense lncRNAs are a subtype of lncRNA molecules transcribed from the opposite DNA strands from those producing the sense transcripts of protein-coding and non-protein-coding genes; they may partly overlap with sense RNAs^12^. Recently, the antisense lncRNA AGAP2-AS1 has been found to act as an oncogene promoting lung cancer cell proliferation, invasion, and migration, and inhibiting apoptosis by repressing the transcription of the tumor-suppressor LATS2 and KLF2^13^. However, the roles of antisense lncRNAs in lung cancer diagnosis and malignant processes, and the mechanism of interaction between antisense lncRNAs and associated genes in lung cancer remain largely unknown.

In our previous work, we identified differentially expressed lncRNAs in patients with lung cancer by using a lncRNA array and identified the circulating lncRNA XLOC_009167 as a diagnostic biomarker for prediction of lung cancer^14^. Among the differentially expressed lncRNAs, DPP10-AS1 is an intergenic antisense lncRNA 744-nt in length, which is located on chromosome 2q14.1. However, the biological function of DPP10-AS1 in lung cancer is unknown. In this study, we found that DPP10-AS1 upregulation was significantly correlated with higher serum CYFRA21-1, larger tumor size, and advanced TNM stage, and was predictive of poor prognosis in patients with lung cancer. Further assays revealed that DPP10-AS1 promoted cell proliferation and cell cycle progression, and inhibited apoptosis by regulating its target gene DPP10. Our results suggest that DPP10-AS1 may be an oncogenic lncRNA that participates in lung cancer development and aggressive progression. In addition, DPP10-AS1 has the potential to serve as a new biomarker for lung cancer diagnosis and therapy.

## Materials and methods

### Patients and tissue preparation

From January 2014 to December 2017, primary lung cancer tissue specimens and cancer-adjacent tissues were collected from patients who underwent radical surgery for lung cancer or palliative resection of lung cancer at the Affiliated Hospital of Ningbo University School of Medicine and Ningbo Medical Center Lihuili Eastern Hospital. The cancer-adjacent tissues were required to be at least 5 cm from the edges of the cancer tissues. We collected 94 specimens from patients who were diagnosed with primary lung cancer and were not treated with preoperative radiotherapy, chemotherapy, targeted therapy, or immunotherapy. The general clinical information and detailed pathological records were collected. Written informed consent was obtained from all patients. The studies were approved by the Clinical Research Ethics Committee of Ningbo University School of Medicine and were conducted in accordance with the Declaration of Helsinki (approval No. NBUSM20151012). The overall survival (OS) of these patients was followed for a median period of 20 months. OS was calculated from the date of surgery to the date of mortality or the last follow-up. Recurrence-free survival (RFS) was calculated from the date of surgery to the date of first recurrence or the last follow-up.

### Cell culture and transient transfection

The normal human bronchial epithelial cell line BEAS-2B and the human lung cancer cell lines A549, H446, SPC-A1, and NCI-H1299 were obtained from a cell bank at the Chinese Academy of Sciences (Shanghai, China). All human lung cancer cell lines were cultured in RPMI-1640 medium (HyClone, Logan, UT, USA) with 10% fetal bovine serum (PAN, Aidenbach, Germany). The cells were maintained in a humidified chamber/incubator containing 5% CO_2_ at 37 °C. BEAS-2B was maintained in Dulbecco’s modified Eagle’s medium (Gibco, Grand Island, NY, USA) supplemented with 10% FBS. All oligonucleotides and plasmids were transfected into SPC-A1 and NCI-H1299 cells with Lipofectamine 2000 Transfection Reagent (Invitrogen, Carlsbad, CA, USA) according to the manufacturer’s protocol. The transfected cells were harvested at 24 or 48 h after transfection. The siRNA oligonucleotides were synthesized by GenePharma (Shanghai, China).

### RNA extraction and quantitative reverse transcription polymerase chain reaction (qRT-PCR)

Total RNA was extracted with TRIzol reagent (Invitrogen, Carlsbad, CA, USA), and cDNA synthesis was then performed with a PrimeScript™ II 1st strand cDNA Synthesis Kit (TaKaRa, Otsu, Japan) according to the standard protocol. qRT-PCR was subsequently performed with SYBR Premix Ex Taq™ II (TaKaRa, Otsu, Japan) according to the manufacturer’s instructions, and was run on an Mx3005P PCR device (Stratagene, La Jolla, CA, USA). The results were normalized to levels of β-actin mRNA. The comparative 2^−ΔΔCt^ method was used for relative quantification and statistical analysis. To account for technical variability, the assays was performed in triplicate for each case.

### Recombinant plasmid construction

The sequences of DPP10-AS1 and DPP10 were amplified by PCR from the genomic DNA of SPC-A1 or NCI-H1299 cell lines, and were cloned into linearized pcDNA 3.1 plasmid (Biogle, Changzhou, China), as described in our previous work^15^.

### Protein isolation and Western blot

Cells were first lysed in RIPA protein extraction regent (Beyotime, Shanghai, China) plus 1 mM PMSF protease inhibitor (Beyotime, Shanghai, China). Protein concentrations were measured with a BCA protein assay kit (Abcam, Cambridge, MA, USA). Western blot was performed as previously described^16^. Briefly, equal amounts (40 μg) of protein were separated by 8% SDS-PAGE and then transferred onto polyvinylidene fluoride membranes (Millipore, Billerica, MA, USA). Subsequently, the membranes were blocked with TBS/0.1% Tween-20 supplemented with 5% skimmed milk for 1 h at room temperature and then incubated with primary antibodies against DPP10 (1:3,000) or β-actin (1:5,000) at 4 °C overnight, washed with TBS containing 0.1% Tween-20, and then incubated with secondary antibodies. The primary antibodies were anti-β-actin antibody (sc-25778, Santa Cruz, Dallas, TX, USA) and anti-DPP10 antibody (ab-111985, Abcam, Cambridge, MA, USA). Finally, the membranes were incubated with the secondary antibodies, rabbit anti-goat IgG-HRP BA-1060 (Boster, Wuhan, China) and goat anti-mouse IgG BA-1050 (Boster, Wuhan, China), and visualized with an infrared imaging system (Li-COR, Lincoln, NE, USA). The immunoreactive bands were quantified by densitometry with ImageJ software when necessary.

### Nuclear and cytoplasmic RNA fractionation analysis

Nuclear and cytosolic fractions were separated with a PARIS kit (Am1921, Thermo Fisher Scientific, Waltham, MA, USA) according to the manufacturer’s instructions. Then the expression levels of β-actin, U6, and DPP10-AS1 in the nuclei and cytoplasm in SPC-A1 cells were detected with qRT-PCR assays.

### RNase protection assays

pcDNA3.1-DPP10-AS1, pcDNA3.1, siNC, and siDPP10-AS1 were co-transfected into SPC-A1 and NCI-H1299 cells. Forty-eight hours later, the RNA was extracted from the cells, and then treated with RNase A + T (Thermo Fisher Scientific, Waltham, MA, USA) for 1 h at 37 °C. Single-stranded RNA was digested with RNase A + T, and the remaining double-stranded RNA was extracted and analyzed with real-time qPCR.

### mRNA stability assays

SPC-A1 and NCI-H1299 cells at a density of 1 × 10^5^/mL were grown in a 35-mm cell culture dish and then cultured overnight to allow for attachment. The cells were exposed to 2 μg/mL of actinomycin D (Sigma-Aldrich, St. Louis, MO, USA) at the indicated time points. Then the cells were collected, and total RNA was extracted for reverse transcription. The stability of DPP10 mRNA was analyzed with RT-qPCR by using cDNA as a template.

### DNA methylation analysis

The methylation profiles of the DPP10 and DPP10-AS1 promoter regions were evaluated with the bisulfite amplicon sequencing method. Briefly, genomic DNA was extracted from lung cancer cells, or paired lung cancer and non-cancer tissues with a genomic DNA extraction kit (Generay, Shanghai, China). The DNA was subsequently bisulfite-converted with EZ DNA Methylation according to the manufacturer’s instructions (Zymo Research, Irvine, CA, USA). A PCR library was generated from the amplicons by using an Illumina TruSeq Nano DNA sample pre kit (Illumina, San Diego, CA, USA). The prepared library was sequenced on a MiSeq system (Illumina, San Diego, CA, USA) with 300-bp paired-end reads. A map and methylation level profile for each CpG position in the DPP10-AS1 and DPP10 promoter region were generated with BS-seeker2 according to the Hg38 reference sequence.

### MTT assays

Briefly, SPC-A1 and NCI-H1299 cells were seeded in 96-well plates at an initial concentration of 5 × 10^3^ cells/well in RPMI-1640 supplemented with 10% FBS. Cells were allowed to grow for 24, 48, 72, or 96 h. Then 20 μL of 5 mg/mL MTT (Sigma-Aldrich, St. Louis, MO, USA) was added into each well, and cells were cultured at 37 °C for 4 h. After the incubation, cells were lysed with 150 μL DMSO. Formazan crystals were dissolved in dimethyl sulfoxide. A microplate reader (Labsystems, Vantaa, Finland) was used to measure the OD values at a wavelength of 490 nm. Each experimental group contained 3 replicate wells, and the experiment was repeated 3 times.

### Colony formation assays

After transfection with siRNAs or constructed plasmids, cells were collected and resuspended in cell culture medium. Five hundred cells were seeded into 6-well plates and cultured at 37 °C with 5% CO_2_. After incubation for 13 days, cell colonies were washed with 1× PBS 3 times. The colonies were fixed with ice-cold methanol and stained with 0.1% crystal violet at room temperature for 10 min.

### Cell cycle analysis

Cells were cultured in 6-well plates overnight and then transfected with siRNAs or constructed plasmids with Lipofectamine 2000 after 24 h of serum starvation for. At 24 h after transfection, the cells were collected and washed with cold 1× PBS. Next, the cells were rewashed with cold 1× PBS and incubated with PI/RNase staining buffer (Multisciences, Hangzhou, China). Cells were analyzed with a FACSCalibur flow cytometer (BD, Franklin Lakes, NJ, USA).

### Cell apoptosis assays

Cell apoptosis was measured with an Annexin V-FITC/PI Apoptosis Detection Kit (Multisciences, Hangzhou, China). In brief, cells were seeded in 6-well plates 1 day before transfection. After 24 h, the cells were collected and washed with cold 1× PBS. Before analysis, the cells were resuspended in 500 μL binding buffer plus 5 μL PI and 5 μL FITC-conjugated anti-Annexin V antibody. After incubation in the dark for 15 min at room temperature, cells were analyzed with a FACSCalibur flow cytometer equipped with Cell Quest software (BD, Franklin Lakes, NJ, USA).

### *In vivo* tumorigenesis assays

The experiments were performed in accordance with guidelines approved by the Laboratory Animal Ethical Committee at Ningbo University (approval No. NBULA20170915). Four-week-old male nude mice were purchased from Shanghai SLAC Laboratory Animals Co., Ltd (Shanghai, China). We screened for lung cancer cells with stable expression of DPP10-AS1 by using geneticin (G418), and injected 5 × 10^6^ experimental cells or empty vector cells into mice for analysis of subcutaneous tumor formation (8 mice per group). The tumor dimensions were measured every 2 days; after 4 weeks, the mice were sacrificed by cervical dislocation, and the tumors were excised and weighed. The tumor volume was calculated with the formula (length × width^2^)/2.

### Statistical analysis

The statistical analyses were performed in GraphPad 7.0 (GraphPad, San Diego, CA, USA). Data are presented as mean ± SD from at least 3 separate experiments. The differences between DPP10-AS1 or DPP10 expression levels in lung cancer tissues and pair-matched noncancerous tissues were compared with the Wilcoxon signed-rank test. The chi-squared test (χ^2^ test) was used to evaluate the relationship between the clinicopathological features and DPP10-AS1 expression. Two sample comparisons were performed with Student’s *t*-test for equal or unequal variance or Pearson correlation test. Mann-Whitney U test was used to assess the differential expression levels of DPP10-AS1 and DPP10 in patient cohorts. The relationship between DPP10-AS1 and DPP10 expression was analyzed with Pearson’s correlation. For patients with different levels of DPP10-AS1 expression, the survival curves were plotted with the Kaplan-Meier method and compared with log-rank tests. Multivariate survival analysis was performed on all parameters found to be significant in univariate analysis by using the Cox regression model. For all analyses, a *P*-value < 0.05 was considered statistically significant. All *P*-values are 2 sided.

## Results

### DPP10-AS1 is upregulated in lung cancer and predicts poor prognosis in patients

**Figure 1:**
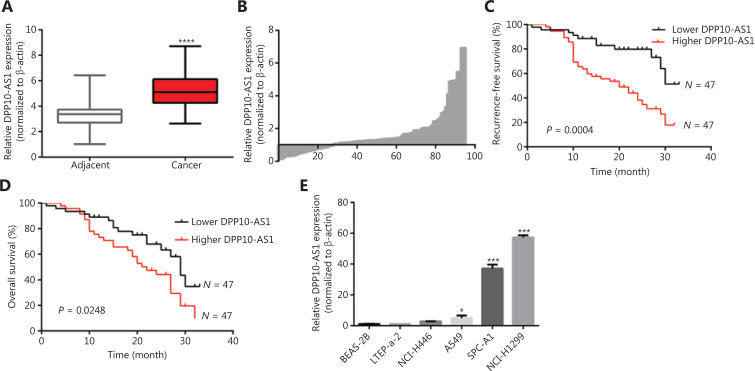
The upregulation of DPP10-AS1 predicts poor prognosis in patients with lung cancer. (A) DPP10-AS1 expression in lung cancer tissues and corresponding noncancerous lung tissues was measured by qRT-PCR and normalized to β-actin. The horizontal lines in the box plots represent the medians. The boxes represent the interquartile ranges, and the whiskers represent percentiles 2.5 and 97.5. Statistical differences between groups were compared with the Wilcoxon signed-rank test (*n* = 94, *P* < 0.0001). (B) The ratio between the relative quantification of DPP10-AS1 expression in lung cancer tissues and paired adjacent noncancerous lung tissues of each case. Kaplan-Meier survival analyses of the correlation between DPP10-AS1 expression levels and recurrence-free survival (C) or overall survival (D) in 94 patients with lung cancer. The median expression level was used as a cutoff. Statistical significance was analyzed with the log-rank test. (E) DPP10-AS1 expression in a normal lung epithelial cell line (BEAS-2B) and in 5 lung cancer cell lines (NCI-H446, A549, LTEP-a-2, SPC-A1, and NCI-H1299). Data are shown as mean ± standard error from at least 3 independent experiments. *P < 0.05, ***P < 0.001, ****P < 0.0001.

To confirm the expression of DPP10-AS1 in lung cancer tissues, we performed qRT-PCR to detect DPP10-AS1 in 94 pairs of lung cancer tissues and adjacent noncancerous tissues. The expression of DPP10-AS1 in tumor tissues from patients with lung cancer was significantly higher than that in corresponding normal tissues (*P* < 0.0001, **Figure 1A**). More specifically, 72% (68/94) of patients with lung cancer showed higher DPP10-AS1 levels in tumor tissues than in adjacent noncancerous tissues (**Figure 1B**). To further investigate the association between DPP10-AS1 expression and clinicopathological characteristics, we divided 94 lung cancer samples into 2 subgroups according to the median relative DPP10-AS1 expression ratio: a high DPP10-AS1 group (*n* = 47, DPP10-AS1 ratio ≥ median ratio) and a low DPP10-AS1 group (*n* = 47, DPP10-AS1 ratio < median ratio). Correlation regression analysis showed that high DPP10-AS1 expression in patients with lung cancer was closely associated with high serum CYFRA21-1 levels (*P* = 0.014), large tumor size (*P* = 0.0079), and advanced TNM stage (*P* = 0.0406). However, age (*P* = 0.3279), gender (*P* = 0.0988), tumor number (*P* = 0.1438), microvascular invasion (*P* = 0.4532), and smoking history (*P* = 0.2049) were not correlated with DPP10-AS1 expression (**Supplementary Table S1**). To further evaluate the prognostic value of DPP10-AS1 in patients with lung cancer, we analyzed the association between DPP10-AS1 expression and survival duration by using Kaplan-Meier analysis with the log-rank test. The results revealed that patients with lung cancer with higher DPP10-AS1 expression had a significantly poorer RFS than patients with lower DPP10-AS1 expression (log rank = 9.329, *P* = 0.0025, **Figure 1C**). Similarly, in the patients with lung cancer, higher DPP10-AS1 expression predicted a poorer OS than lower DPP10-AS1 expression (log rank = 9.333, *P* = 0.036, **Figure 1D**). Cox proportional hazard regression analysis further showed that high DPP10-AS1 expression in lung cancer tissues was an independent predictor of poorer RFS (**Supplementary Table S2**) and OS (**Supplementary Table S3**). These results indicated that DPP10-AS1 expression is upregulated in lung cancer tissues and predicts poor prognosis in patients with lung cancer.

We also measured the DPP10-AS1 expression levels in 5 lung cancer cell lines and normal human bronchial epithelial cells (BEAS-2B) by qRT-PCR. As shown in **Figure 1E**, the relative DPP10-AS1 expression in lung cancer cells (A549, SPC-A1, and NCI-H1299) was significantly upregulated (5–57-fold change), as compared with that in human normal bronchial epithelial cells. Thus, SPC-A1 and NCI-H1299 cell lines were selected for subsequent assays. Collectively, these results indicated that DPP10-AS1 is elevated *in vivo* and* in vitro*, and can be considered an independent prognostic factor of outcomes in patients with lung cancer.

### DPP10-AS1 promotes lung cancer cell proliferation *in vitro* and facilitates tumor growth in a xenograft animal model

**Figure 2:**
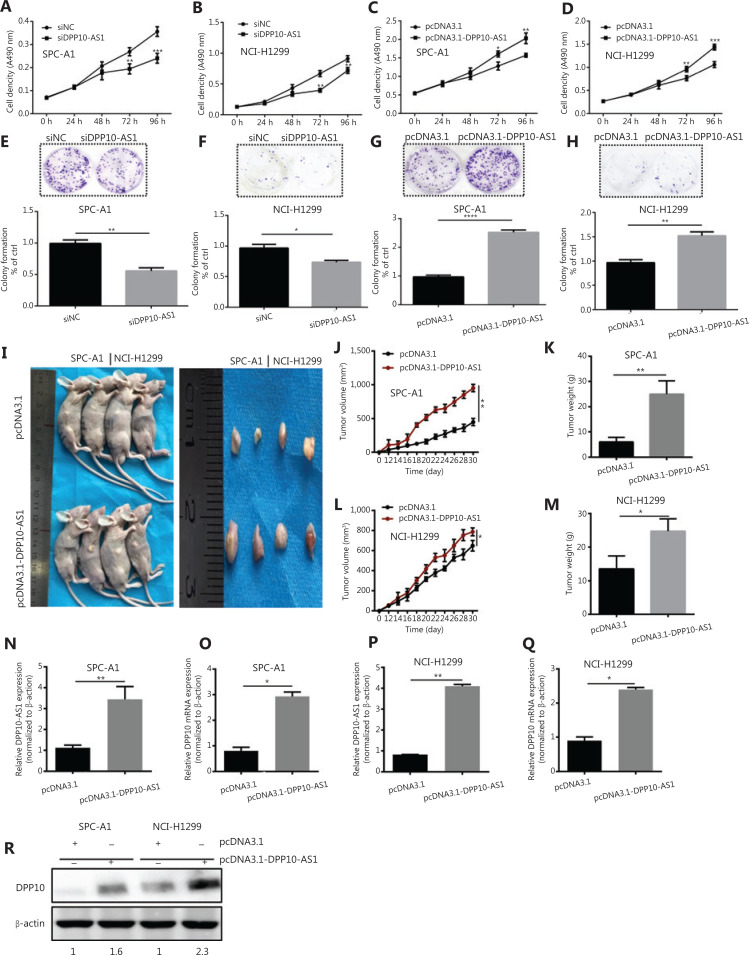
DPP10-AS1 promotes lung cancer cell proliferation *in vitro* and tumor growth *in vitro*. MTT assays were performed to determine the viability of lung cancer cells treated with siDPP10-AS1 in SPC-A1 (A) and NCI-H1299 (B) cells, and with pcDNA3.1-DPP10-AS1 in SPC-A1 (C) and NCI-H1299 (D) cells. Colony formation assays were used to detect the proliferation ability of lung cancer cells after transfection with siDPP10-AS1 in SPC-A1 (E) and NCI-H1299 (F) cells, and pcDNA3.1-DPP10-AS1 in SPC-A1 (G) and NCI-H1299 (H) cells. Lung cancer cells overexpressing pcDNA3.1-DPP10-AS1 were injected subcutaneously into nude mice to demonstrate xenograft tumor growth (I). Analysis of tumor volume (J) and tumor weight (K) in SPC-A1 cells with overexpression of pcDNA3.1-DPP10-AS1. Analysis of tumor volume (L) and tumor weight (M) in NCI-H1299 cells with overexpression of pcDNA3.1-DPP10-AS1. RT-qPCR analysis of lncRNA DPP10-AS1 (N) and DPP10 mRNA (O) expression in tumor tissues after injection of SPC-A1 cells. RT-qPCR analysis of lncRNA DPP10-AS1 (P) and DPP10 mRNA (Q) expression in tumor tissues after injection of NCI-H1299 cells. Western blot assays of DPP10 protein in tumor tissues overexpressing lncRNA DPP10-AS1 and empty plasmid (R). Colonies were counted and captured. The bar charts statistically compare the differences in colony formation in each experimental group compared with the corresponding control. Values are shown as the mean ± SD in 3 independent experiments. *P < 0.05, **P < 0.01, ***P < 0.001, ****P < 0.0001.

Because the significant upregulation of DPP10-AS1 in lung cancer specimens was associated with larger tumor size, we explored the effect of DPP10-AS1 on lung cancer cell growth. To regulate DPP10-AS1 expression in lung cancer cells, the endogenous expression of DPP10-AS1 was inhibited by transfection of siRNA and overexpressed through transfection of pcDNA3.1-DPP10-AS1. DPP10-AS1 was downregulated up to 50%–60% by siRNA knockdown (**Supplementary Figure S1A**) and was upregulated up to 250–300 fold by overexpression (**Supplementary Figure S1B**). MTT assays indicated that knockdown of endogenous DPP10-AS1 expression dramatically inhibited the growth of SPC-A1 and NCI-H1299 cells (**Figure 2A, 2B**). In contrast, overexpression of DPP10-AS1 significantly promoted cell growth in both cell lines (**Figure 2C, 2D**). Further colony formation assays showed that downregulation of DPP10-AS1 significantly inhibited colony formation in both SPC-A1 and NCI-H1299 cells (**Figure 2E, 2F**). In contrast, overexpression of DPP10-AS1 promoted colony formation in both cell lines (**Figure 2G, 2H**). To further confirm the effect of DPP10-AS1 on lung tumor growth *in vivo*, we screened 2 lung cancer cell lines (SPC-A1 and NCI-H1299) stably expressing DPP10-AS1 by using geneticin (G418) and subcutaneously injected them into nude mice to establish a xenograft tumor model (**Figure 2I**). Overexpression of DPP10-AS1 promoted tumor growth in terms of tumor volume (**Figure 2J**) and weight (**Figure 2K**) in the xenograft animal model with injection of SPC-A1 cells. Similar effects were found in the NCI-H1299 cell-derived xenograft animal model (**Figure 2L, 2M**). In addition, compared with the negative control (pcDNA3.1), pcDNA3.1-DPP10-AS1 resulted in an increase in the lncRNA DPP10-AS1 (**Figure 2N**) and *DPP10* mRNA (**Figure 2O**) in SPC-A1 cell-derived tumor tissues. Similar results were obtained in NCI-H1299 cell-derived tumor tissues (**Figure 2P, 2Q**). At the protein level, overexpression of the lncRNA DPP10-AS1 promoted cognate DPP10 protein expression (**Figure 2R**). Thus, the data suggested that DPP10-AS1 promotes lung cancer cell growth and colony formation *in vitro*, and facilitates lung tumor growth *via* upregulation of DPP10 protein in a xenograft animal model.

### DPP10-AS1 promotes cell cycle progression and represses apoptosis in lung cancer cells

**Figure 3:**
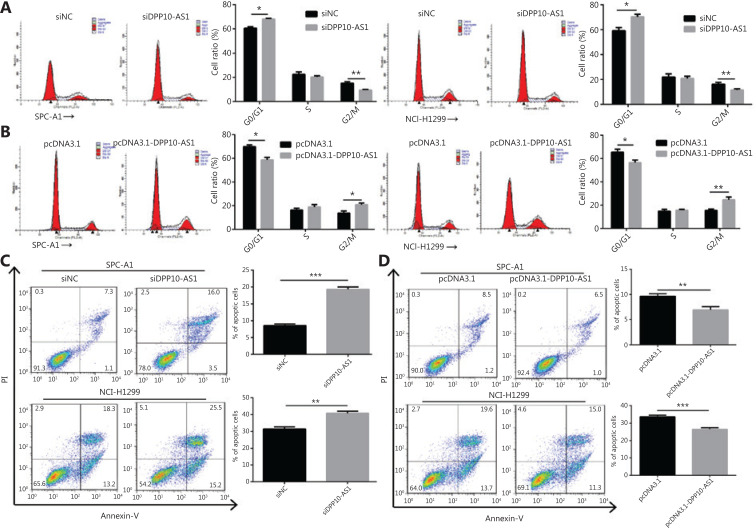
DPP10-AS1 promotes cell cycle progression and inhibits apoptosis in lung cancer cells. SPC-A1 and NCI-H1299 lung cancer cells were treated with siDPP10-AS1 (A) or pcDNA3.1-DPP10-AS1 (B) and then were analyzed for cell cycle by flow cytometry. After the knockdown of DPP10-AS1 (C) or overexpression of DPP10-AS1 (D), lung cancer cell apoptosis was analyzed by flow cytometry with Annexin V/PI staining. The data are presented as the mean ± SD (*n* = 3), and *P < 0.05, **P < 0.01, ***P < 0.001.

To probe the potential mechanisms through which DPP10-AS1 enhances lung cancer cell proliferation, we assessed the cell cycle and apoptosis in SPC-A1 and NCI-H1299 cells after DPP10-AS1 knockdown or overexpression. Flow cytometric cell cycle assays demonstrated that knockdown of DPP10-AS1 led to a significant accumulation of cells in G0/G1 phase and a significant decrease in cells in G2/M-phase in both cell lines (**Figure 3A**). Conversely, overexpression of DPP10-AS1 resulted in a marked decrease in the G0/G1 cell population and an increase in cells in G2/M phase in both cell lines (**Figure 3B**). Moreover, the cell apoptosis assays indicated that knockdown of DPP10-AS1 significantly increased early and late apoptosis in both SPC-A1 and NCI-H1299 cells (**Figure 3C**). In contrast, overexpression of DPP10-AS1 dramatically decreased early and late apoptosis in both cell lines (**Figure 3D**). Collectively, our results indicated that the DPP10-AS1-induced lung cancer cell growth appears to be mediated by cell cycle arrest at G2/M-phase and repression of apoptosis.

### DPP10-AS1 positively regulates *DPP10* gene expression

**Figure 4:**
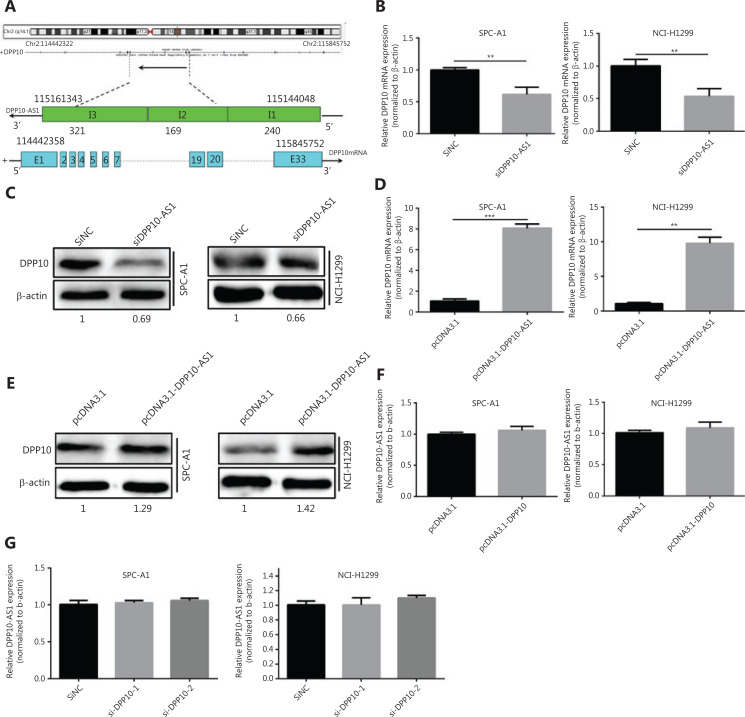
DPP10-AS1 positively regulates *DPP10*. (A) Genetic structure diagram of DPP10-AS1. (B) Effect of DPP10-AS1 knockdown on *DPP10* mRNA. (C) Effect of DPP10-AS1 overexpression on DPP10 protein. (D) Effect of DPP10 overexpression on *DPP10* mRNA. (E) Effect of DPP10-AS1 overexpression on DPP10 protein. (F) Effect of DPP10 overexpression on DPP10-AS1. (G) Effect of DPP10 knockdown on DPP10-AS1. Data are shown as mean ± SD from 3 independent experiments. **P < 0.01, ***P < 0.001.

DPP10-AS1 is a conserved 744-nt RNA transcribed in the antisense direction from the protein-coding gene *DPP10* (2q14.1) (**Figure 4A**). To confirm the regulatory relationship between DPP10-AS1 and *DPP10*, we detected the expression levels of DPP10-AS1 and its sense-cognate gene *DPP10*. Knockdown of DPP10-AS1 decreased *DPP10* gene expression at the mRNA (**Figure 4B**) and protein (**Figure 4C**) levels in both SPC-A1 and NCI-H1299 cells. In contrast, overexpression of DPP10-AS1 enhanced *DPP10* mRNA (**Figure 4D**) and protein (**Figure 4E**) expression in both lung cancer cell lines. To explore the effect of *DPP10* on DPP10-AS1 expression in lung cancer cells, we used *DPP10* siRNA and pcDNA3.1-DPP10 to inhibit and overexpress *DPP10*, respectively. The siRNA decreased the endogenous *DPP10* gene expression at the mRNA (**Supplementary Figure S2A**) and protein (**Supplementary Figure S2B**) levels in both SPC-A1 and NCI-H1299 cells. In contrast, overexpression of *DPP10* mediated by transfection with pcDNA3.1-DPP10 increased *DPP10* gene expression at the mRNA (**Supplementary Figure S2C**) and protein (**Supplementary Figure S2D**) levels in both lung cancer cell lines. However, neither overexpression nor knockdown of *DPP10* affected the expression of DPP10-AS1 in lung cancer cells (**Figure 4F, 4G**). These results suggested that the expression of the *DPP10* gene is positively regulated by DPP10-AS1.

### DPP10-AS1 and *DPP10* are coordinately upregulated in lung cancer cells and tissues

**Figure 5:**
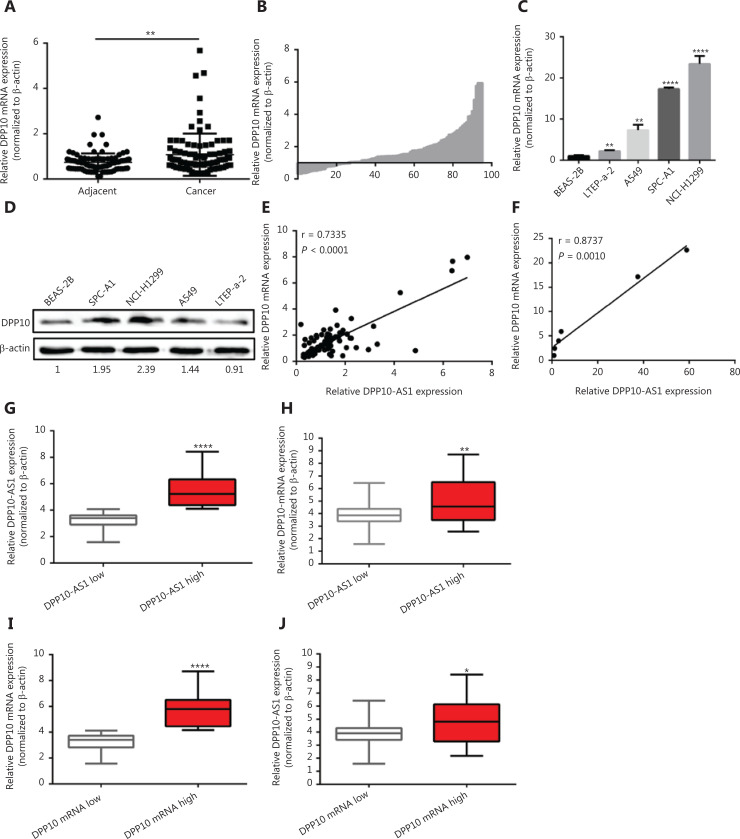
DPP10-AS1 and DPP10 are coordinately upregulated in lung cancer. (A) Difference in expression levels of *DPP10* mRNA between lung cancer tissues and adjacent cancerous tissues. β-actin served as the internal control for normalization. The statistical difference was analyzed with Wilcoxon signed-rank test. (B) The ratio between the relative quantification of *DPP10* mRNA expression in lung cancer tissues *vs.* paired adjacent noncancerous lung tissues for each case. qRT-PCR (C) and Western blot (D) analysis of the relative expression of *DPP10* mRNA and protein level in lung cell lines. The correlation between DPP10-AS1 and *DPP10* mRNA in lung cancer tissues (E) and cell lines (F). Data were subjected to Pearson correlation analysis. (G, H) According to the expression of DPP10-AS1, the qRT-PCR data from 94 pairs of clinical samples were classified as DPP10-AS1 high and DPP10-AS1 low. The relative expression of *DPP10* mRNA and DPP10-AS1 is compared in box plots. (I, J) According to the expression of DPP10, the qRT-PCR data from 94 pairs of clinical samples were classified as *DPP10* high and *DPP10* low. The relative expression of DPP10-AS1 and *DPP10* is compared in box plots. *P < 0.05, **P < 0.01, ****P < 0.0001.

Given the positive regulation of *DPP10* by DPP10-AS1, we also detected the expression of *DPP10* mRNA in the same cohort of 94 paired lung cancer tissue samples by using qRT-PCR. DPP10 expression in lung cancer tissues was significantly higher than that in corresponding adjacent tissues (*P* < 0.01, **Figure 5A, 5B**). We also measured the *DPP10* expression in a panel of lung cancer cell lines and found greater *DPP10* mRNA expression of in 4 lung cancer cell lines than in normal human bronchial epithelial cells (BEAS-2B) (**Figure 5C**). Similarly, the protein expression of *DPP10* was confirmed by Western blot in lung cancer cells (**Figure 5D**). Moreover, the relative expression of *DPP10* mRNA was positively correlated with DPP10-AS1 in lung cancer tissues (*r* = 0.7335, *P* < 0.0001, **Figure 5E**) and lung cancer cell lines (*r* = 0.8737, *P* = 0.0010, **Figure 5F**). To further assess the relevance of these findings, we divided the lung cancer specimens into 2 groups: the DPP10-AS1 high group, with higher DPP10-AS1 in tumor tissues than in paired adjacent non-tumor tissues, and the DPP10-AS1 low group, with lower DPP10-AS1 in tumor tissues than in the paired adjacent non-tumor tissues (**Figure 5G**). Notably, the mRNA level of *DPP10* was higher in the DPP10-AS1 high group than the DPP10-AS1 low group (**Figure 5H**). Similarly, we examined *DPP10* mRNA high and *DPP10* mRNA low groups to distinguish the relative expression of *DPP10* mRNA (**Figure 5I**). As expected, the RNA level of DPP10-AS1 was clearly higher in the *DPP10* mRNA high subset than the *DPP10* mRNA low subset (**Figure 5J**). Together, these results indicated that the upregulation of DPP10-AS1 is coordinately correlated with *DPP10* mRNA expression in lung cancer cell lines and in the tissues of patients with lung cancer.

### DPP10-AS1 promotes malignant processes and inhibits apoptosis through upregulating *DPP10* expression

**Figure 6:**
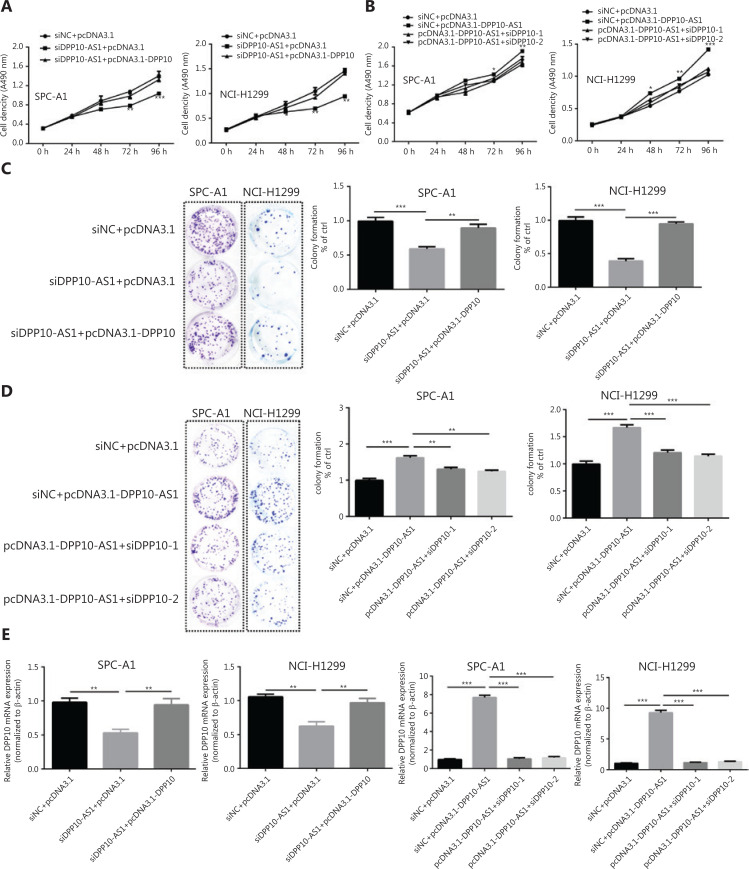
DPP10-AS1 promotes lung cancer cell growth and proliferation through upregulating *DPP10* mRNA expression. (A) Rescue effect of DPP10 overexpression on DPP10-AS1 knockdown-mediated cell growth inhibition in SPC-A1 and NCI-H1299 cells, determined with MTT assays. (B) Rescue effect of DPP10 knockdown on DPP10-AS1 overexpression-mediated cell growth promotion, determined with MTT assays. (C) Colony formation assays were performed to investigate the effects of DPP10-AS1 knockdown and DPP10 overexpression on SPC-A1 and NCI-H1299 cell proliferation. (D) The effects of DPP10-AS1 overexpression and DPP10 knockdown on SPC-A1 and NCI-H1299 cell proliferation were detected with colony formation assays. The number of colonies was counted, and statistical analysis is shown at right. (E) Rescue effects of DPP10 overexpression or depletion on DPP10-AS1-mediated *DPP10* mRNA expression changes. Data are shown as mean ± SD from 3 independent experiments. *P < 0.05, **P < 0.01, ***P < 0.001.

To investigate whether the coordinate upregulation of DPP10-AS1 and *DPP10* might affect malignant processes of lung cancer cells, we detected cell behavior after overexpression of DPP-10 as well as knockdown of DPP10-AS1. In both SPC-A1 and NCI-H1299 cells, MTT assays showed that knockdown of DPP10-AS1 inhibited lung cancer cell growth, whereas simultaneous overexpression of *DPP10* abolished the suppressive effect mediated by knockdown of DPP10-AS1 (**Figure 6A**). Conversely, overexpression of DPP10-AS1 promoted lung cancer cell growth, whereas simultaneous knockdown of *DPP10* abrogated the promotion effect mediated by overexpression of DPP10-AS1 (**Figure 6B**). Furthermore, colony formation assays showed that DPP10-AS1 downregulation inhibited the colony formation of lung cancer cells, and the overexpression of DPP10 rescued colony formation ability in both SPC-A1 and NCI-H1299 cell lines (**Figure 6C**). In contrast, overexpression of DPP10-AS1 promoted colony formation in SPC-A1 and NCI-H1299 cells, whereas knockdown of *DPP10* abolished this promotion effect (**Figure 6D**). At the RNA level, overexpression of *DPP10* rescued the suppressive effect of downregulation of DPP10-AS1 on *DPP10* mRNA, and knockdown of DPP10 abolished the positive regulatory effect of DPP10-AS1 on *DPP10* mRNA in both cell lines (**Figure 6E**). These results demonstrated that DPP10-AS1 affects cell growth and proliferation through regulating *DPP10* mRNA expression.

**Figure 7:**
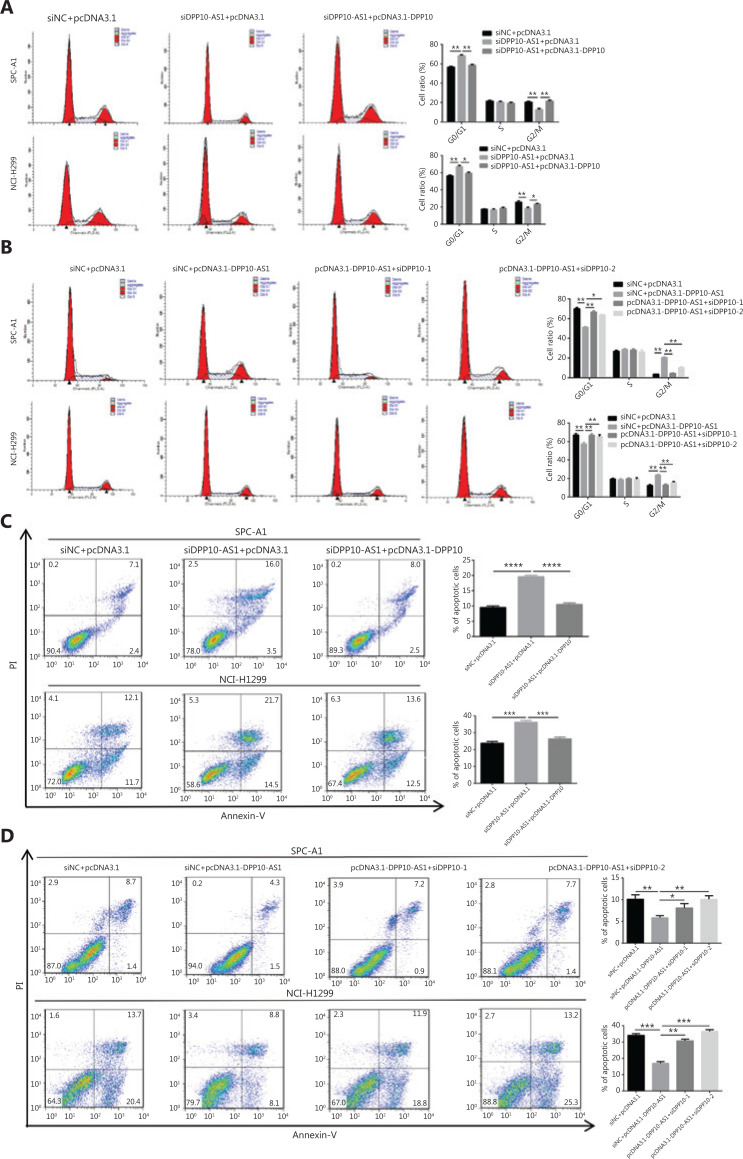
DPP10-AS1 promotes cell cycle progression and inhibits apoptosis by upregulating *DPP10* mRNA. (A) Effects of DPP10-AS1 knockdown and DPP10 overexpression on the SPC-A1 and NCI-H1299 cell cycle, as assessed by flow cytometry. (B) Effects of DPP10-AS1 overexpression and DPP10 knockdown on the SPC-A1 and NCI-H1299 cell cycle, as assessed by flow cytometry. (C) Effects of DPP10-AS1 knockdown and DPP10 overexpression on SPC-A1 and NCI-H1299 cell apoptosis, as assessed by flow cytometry with Annexin V/PI staining. (D) Effects of DPP10-AS1 overexpression and DPP10 knockdown on SPC-A1 and NCI-H1299 cell apoptosis, as assessed by flow cytometry with Annexin V/PI staining. Data are shown as mean ± SD from 3 independent experiments. *P < 0.05, **P < 0.01, ***P < 0.001, ****P < 0.0001.

In addition, cell cycle analysis showed that knockdown of DPP10-AS1 induced cell cycle arrest at G0/G1 phase and decreased the cell population in G2/M phase; this effect was abolished by the overexpression of *DPP10* in SPC-A1 and NCI-H1299 cells (**Figure 7A**). Furthermore, overexpression of DPP10-AS1 decreased the cell population in G0/G1 phase and resulted in cell cycle arrest at G2/M phase. However, this effect was also abolished by the depletion of *DPP10* (**Figure 7B**). Furthermore, apoptosis analysis showed that overexpression of *DPP10* abolished the DPP10-AS1 knockdown-mediated increase in early and late apoptotic cells in both lung cancer cell lines (**Figure 7C**). Conversely, the depletion of *DPP10* rescued the DPP10-AS1 overexpression-mediated decrease in early and late apoptotic cells in lung cancer cells (**Figure 7D**). Collectively, the data suggested that DPP10-AS1 promotes cell growth and proliferation, induces cell cycle arrest, and inhibits apoptosis through upregulating *DPP10* gene expression in lung cancer cells.

### DPP10-AS1 is associated with *DPP10* mRNA but does not enhance *DPP10* mRNA stability

**Figure 8:**
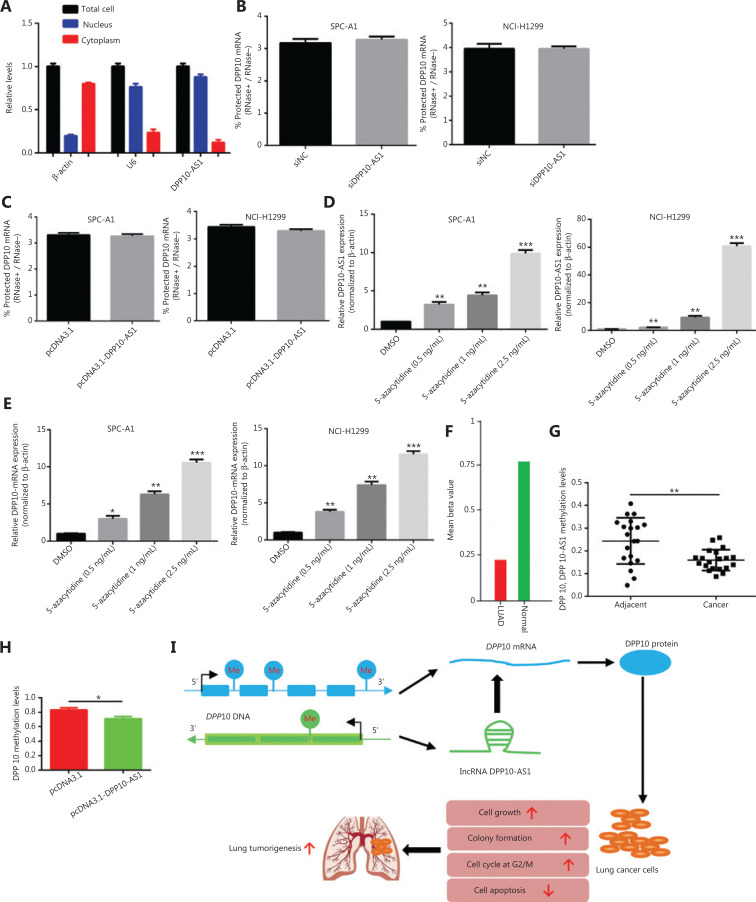
DPP10-AS1 does not increase *DPP10* mRNA stability, but both are regulated by epigenetic methylation. (A) qRT-PCR analysis of DPP10-AS1 in the nucleus and cytoplasm, as determined with cytoplasmic and nuclear extract isolation assays. (B) qRT-PCR analysis of *DPP10* mRNA levels in SPC-A1 and NCI-H1299 cells cotransfected with siDPP10-AS1, as determined with RNase protection assays. (C) qRT-PCR analysis of *DPP10* mRNA levels in SPC-A1 and NCI-H1299 cells cotransfected with pcDNA3.1-DPP10-AS1, as determined with RNase protection assay. (D) qRT-PCR analysis of DPP10-AS1 levels in SPC-A1 and NCI-H1299 cells treated with different doses of the DNA methyltransferase inhibitor 5-azacytidine. (E) qRT-PCR analysis of *DPP10* mRNA levels in SPC-A1 and NCI-H1299 cells treated with different doses of the DNA methyltransferase inhibitor 5-azacytidine. (F) Hypomethylation of *DPP10* in patients with lung cancer (LUAD) compared with healthy controls, according to the MethHC database. (G) DNA methylation analysis of CpG islands of DPP10 and DPP10-AS1 genes in lung cancer tissues. (H) The effect of DPP10-AS1 overexpression on DPP10 methylation in SPC-A1 cells. (I) The working model in which the lncRNA DPP10-AS1 promotes lung carcinoma malignant processes through epigenetic activation of DPP10. *P < 0.05, **P < 0.01, ***P < 0.001.

To study whether DPP10-AS1 might enhance the stability of its sense-cognate gene *DPP10*, we determined the nucleoplasmic localization of DPP10-AS1. Nuclear and cytoplasmic fractionation analysis showed that DPP10-AS1 was mainly located in the nuclei (**Figure 8A**). Next, we performed RNase protection assays to examine the RNA duplex formation between DPP10-AS1 and *DPP10* mRNA. *DPP10* mRNA was fully digested, and no difference in protection of *DPP10* mRNA was found after DPP10-AS1 knockdown (**Figure 8B**) or DPP10-AS1 overexpression (**Figure 8C**), thus suggesting that *DPP10* mRNA cannot form an RNA duplex with DPP10-AS1 and protect it from RNase digestion. Further mRNA stability assays showed that actinomycin D treatment induced time-dependent transcription inhibition in both SPC-A1 and NCI-H1299 cells (**Supplementary Figure S3**). These results indicated that DPP10-AS1 associates with *DPP10* mRNA but does not enhance DPP10 mRNA stability through formation of an RNA duplex.

### Hypomethylation of DPP10-AS1 and *DPP10* contributes to their coordinate upregulation in lung cancer

To further reveal the underlying mechanism responsible for the coordinate upregulation of DPP10-AS1 and *DPP10* in lung cancer, we used the DNA methyltransferase inhibitor 5-azacytidine to determine the effect of methylation on the expression of DPP10-AS1 and *DPP10*. The relative expression of DPP10-AS1 was markedly upregulated with increasing 5-azacytidine concentration in both SPC-A1 and NCI-H1299 cells (**Figure 8D**). Likewise, the relative expression of *DPP10* mRNA significantly increased in a 5-azacytidine dose-dependent manner in the same cell lines (**Figure 8E**). These results suggested that hypomethylation of DPP10-AS1 and *DPP10* may contribute to the coordinate upregulation of DPP10-AS1 and *DPP10* in lung cancer. Interestingly, hypomethylation of *DPP10* is present in patients with lung cancer compared with healthy controls, according to the Meth HC database (**Figure 8F**). Similarly, the methylation levels at CpG islands in both DPP10-AS1 and DPP10 genes in lung cancer tissues were lower than those in their adjacent normal tissues (**Figure 8G**); both DPP10-AS1 and DPP10 share 4 common methylation sites (**Supplementary Figure S4**). Furthermore, the methylation levels under DPP10-AS1 overexpression were lower than those in the control group (**Figure 8H**). DPP10-AS1 overexpression led to a decrease in DPP10 methylation, thus suggesting that DPP10-AS1 regulates hypomethylation of DPP10. Together, the data indicated that the coordinate upregulation of DPP10-AS1 and *DPP10* is potentially modulated by their hypomethylation, and the upregulation of DPP10, as positively regulated by DPP10-AS1, is a key event in lung cancer progression (**Figure 8I**).

## Discussion

Mammalian genomes are now widely known to encode numerous lncRNAs^17^. Dysregulation of some lncRNAs has been demonstrated in various type of cancers, including lung cancer^18,19^. However, the functions and mechanisms underlying antisense lncRNAs in lung cancer remain obscure. According to our previous lncRNA microarray data, we identified a novel antisense lncRNA, DPP10-AS1, which is more highly expressed in lung cancer tissues than in adjacent noncancerous tissues. In addition, we explored the role of DPP10-AS1 in lung cancer malignant processes. We found that the upregulation of DPP10-AS1 in cancer tissues was associated with serum CYFRA21-1 level, tumor size, TNM stage, RFS, and OS in patients. Therefore, it may serve as an independent prognostic factor for patients with lung cancer. Moreover, *in vitro* experiments showed that inhibition of DPP10-AS1 repressed lung cancer cell proliferation and induced cell cycle arrest at G1/G0 phase as well as cell apoptosis. Thus, the data confirmed the hypothesis that the novel lncRNA DPP10-AS1 exerts tumor oncogenic activity, thereby promoting malignant processes in lung cancer. We also revealed a new molecular mechanism through which the antisense lncRNA DPP10-AS1 positively regulates its related gene DPP10 *via* epigenetic modification. Therefore, this study provides novel insight into the role of DPP10-AS1 in lung cancer.

Notably, lncRNAs oriented in the antisense direction with respect to protein coding loci on the opposite strand often act as regulators of their related genes^20–23^. Previous studies have shown that natural antisense transcripts play critical roles in various physiological and pathological processes through regulating sense gene promoter activation and controlling mRNA stability and translatability^24–27^. For example, natural antisense transcripts such as TFPI2AS1 and FOXC2-AS1 bind the sense transcripts and alter the stability and expression of the corresponding sense transcripts TFPI2 and FOXC2^28,29^. Although natural antisense transcripts bind the sense transcripts and alter the stability and expression of corresponding sense transcripts, recent studies have shown that upstream antisense transcription may function as an activator of corresponding gene expression^30,31^. Dimitrova et al.^32^ have found that lincRNA p-21 acts as enhancer for the p21 promoter. In our study, DPP10-AS1 was found to be located in the antisense DNA strand of the *DPP10* gene. Therefore, we hypothesized that DPP10-AS1 might regulate DPP10 and contribute to progression of malignant diseases. Further experiments revealed that knockdown of DPP10-AS1 decreased *DPP10* mRNA and protein expression in lung cancer cells, whereas overexpression of DPP10-AS1 increased *DPP10* mRNA and protein expression. Many coding-gene promoters sustain divergent transcription of lncRNA/mRNA gene pairs^30^. Antisense transcription from bidirectional promoters has been shown to be involved in gene regulation^33^. Because of the nuclear location of DPP10-AS1, whether DPP10-AS1 regulates the activity of the *DPP10* promoter requires further investigation. In addition, DPP10 inhibition partially abrogated DPP10-AS1-induced lung cancer cell growth, proliferation, cell cycle arrest at G1/G0 phase, and apoptosis, whereas DPP10 overexpression showed an opposite effect in lung cancer cells. To date, the mechanism underlying DPP10-AS1/DPP10-mediated cell cycle progression and apoptosis has not been reported. We suspect that DPP10-AS1 may regulate DPP10 expression and consequently influence the downstream effectors. These effector molecules may activate or inhibit cell cycle regulation. To reveal the actual mechanism underlying how DPP10-AS1 affects DPP10 expression, we used an RNase protection assay to test the possibility of RNA duplex formation. However, the results showed that DPP10-AS1 did not form RNA duplexes with *DPP10* mRNA and subsequently increase DPP10 mRNA stability. Thus, DPP10-AS1’s promotion of expression of its sense gene, *DPP10*, is probably caused by other modifications or regulations.

DNA methylation is an epigenetic modification that plays a key role in transcriptional regulation and is associated with most human malignancies^34,35^. Genome-wide early hypomethylation appears to affect both protein coding and long noncoding regions of the genome^36,37^. The hypomethylation of noncoding DNA regions results in the overexpression of lncRNA AFAP1-AS1 and correlates with esophageal adenocarcinoma progression^38^. A genome-wide screen has identified the differentially methylated Esrp2-as, which is significantly upregulated in human breast cancer and is associated with poor prognosis^39^. In the current study, we found that both DPP10-AS1 and *DPP10* were coordinately methylated, on the basis of treatment with 5-azacytidine. Interestingly, the *DPP10* methylation level in lung squamous cell carcinoma was significantly lower than that in healthy controls from the MethHC methylation database. Furthermore, computational analysis predicted 3 CpG islands in the promoter of *DPP10*, as well as one CpG island in DPP10-AS1 spanning the transcription initiation site. These results indicate that differential methylation of DPP10-AS1 and *DPP10* may be responsible for their coordinate upregulation in lung cancer.

The recent application of next-generation sequencing to a growing number of cancer transcriptomes has indeed revealed thousands of lncRNAs whose aberrant expression is associated with different cancer types. Among the few that have been functionally characterized, several have been associated with malignant transformation. Notably, these lncRNAs have key roles in gene regulation and thus affect various aspects of cellular behavior, including differentiation, proliferation, invasion, migration, or genomic stability^40^. In addition, the highly expressed lncRNAs function as oncogenes that activate oncogenic signaling pathways and promote carcinogenesis^41–43^. Importantly, the potential value of lncRNAs in cancer diagnosis, prognosis, and targeted therapy has also been shown^14,44,45^. Interestingly, *DPP10* was originally identified as a prognostic marker and a therapeutic gene in patients with cancer^46,47^. However, the potential regulatory mode and carcinogenic mechanism of *DPP10* in cancers remain unclear. Here, we demonstrate that DPP10-AS1 acts as a positive regulator of *DPP10*, which in turn exerts an oncogenic effect. In this study, we evaluated the biological function of DPP10-AS1 in lung cancer. DPP10-AS1 is widely involved in lung cancer pathophysiology by increasing cell growth and proliferation, promoting cell cycle progression, and inhibiting apoptosis. Meanwhile, our data indicated that DPP10-AS1 is an independent prognostic predictor for both overall survival and recurrence-free survival, thus suggesting a potential role of DPP10-AS1 in lung cancer diagnosis and prognosis. However, the molecular mechanism underlying the physical interaction of DPP10-AS1 and the *DPP10* gene requires further investigation for better understanding of lncRNA DPP10-AS1 in lung carcinogenesis.

## Conclusions

In summary, we identified a novel lncRNA, DPP10-AS1, which is highly expressed in lung cancer and whose upregulation predicted poor prognosis in patients with lung cancer. Notably, knockdown of DPP10-AS1 inhibited cell proliferation, promoted cell cycle progression and triggered early and late apoptosis. In contrast, overexpression of DPP10-AS1 showed the opposite effects. Importantly, DPP10-AS1 promoted lung cancer malignant processes by positively regulating *DPP10* gene expression. The coordinate upregulation of DPP10-AS1 and* DPP10* was found to be mediated by epigenetic hypomethylation. Collectively, these findings provide novel insights into DPP10-AS1 as a candidate prognostic biomarker and a potential therapeutic target in lung cancer.
