# Supplementary material for: LncRNA DPP10-AS1 promotes malignant processes through epigenetically activating its cognate gene DPP10 and predicts poor prognosis in lung cancer patients
Source: Cancer Biol Med. 2021 Aug 15;18(3):675–92. doi: 10.20892/j.issn.2095-3941.2020.0136 (PMC8330531; doi:10.20892/j.issn.2095-3941.2020.0136)
Supplement: Supplementary file 1 [file cbm-18-675-s001.pdf]

Supplementary materials

Table S1 Correlations between DPP10-AS1 and clinical characteristics in 94 patients with lung cancer

Characteristics	*n*	DPP10-AS1 levels†		χ^2 text	*P* value
		Low	High		
Total cases	94	47	47		
Gender					
Male	46	27	19	2.725	0.0988
Female	48	20	28		
Age (years)					
≤ 55	34	14	20	0.9570	0.3279
> 55	60	31	29		
Serum CYFRA21-1					
≤ 3.3	50	32	18	6.033	0.0140*
> 3.3	46	17	27		
Tumor number					
Single	72	39	33	2.136	0.1438
Multiple	22	8	14		
Tumor size (cm)					
≤ 5	64	38	26	7.050	0.0079**
> 5	30	9	21		
TNM stage					
I/II	65	35	30	4.191	0.0406*
III	29	9	20		
Microvascular invasion					
Present	36	17	19	0.5626	0.4532
Absent	58	32	26		
Smoking history					
Yes	44	18	26	1.607	0.2049
No	50	27	23		

†Median expression level was used as a cutoff. Low expression of DPP10-AS1 in 94 patients was defined as a value below the 50th percentile, and high expression was defined as a value above the 50th percentile. *P* values were determined with Pearson's chi-square test. **P* < 0.05, ***P* < 0.01.

Table S2 Univariate and multivariate analysis of clinicopathological variables for RFS rates in 94 patients with lung cancer

Variables	Univariate analysis			Multivariate analysis		
	P value	HR	95%CI	*P* value	HR	95%CI
Gender (male *vs.* female)	0.324	1.036	0.575–1.686			
Age (> 55 *vs.* ≤ 55 years)	0.431	1.231	0.787–2.325			
Serum CYFR1 (> 3.3 *vs.* ≤ 3.3 ng/mL)	0.336	0.957	0.794–3.240			
Smoking history (yes *vs.* no)	0.759	1.325	0.885–3.945			
Tumor number (single *vs.* multiple)	0.576	1.695	0.759–5.621			
Microvascular invasion (present *vs.* absent)	0.042	2.168	1.285–4.326			
TNM stage (I/II *vs.* III)	0.036	0.413	0.226–0.894			
Tumor size (> 5 cm *vs.* ≤ 5 cm)	0.021	2.186	1.273–4.536	0.032	2.304	1.219–3.947
DPP10-AS1 expression (higher *vs.* lower)	0.009	2.84	1.363–5.975	0.012	2.446	1.116–5.823

RFS, recurrence-free survival; HR, hazard ratio; CI, confidence interval.

Table S3 Univariate and multivariate analysis of clinicopathological variables for OS rates in 94 patients with lung cancer

Variables	Univariate analysis			Multivariate analysis		
	P value	HR	95%CI	*P* value	HR	95%CI
Gender (male *vs.* female)	0.527	1.196	0.687–2.080			
Age (> 55 *vs.* ≤ 55 years)	0.921	1.001	0.976–1.028			
Serum CYFR1 (> 3.3 *vs.* ≤ 3.3 ng/mL)	0.405	0.759	0.398–1.450			
Smoking history (yes *vs.* no)	0.302	1.526	0.684–3.405			
Microvascular invasion (present *vs.* absent)	0.546	1.156	0.789–2.123			
Tumor number (single *vs.* multiple)	0.865	1.191	0.159–8.932			
Tumor size (> 5 cm *vs.* ≤ 5 cm)	0.008	3.382	1.374–8.330	0.652	1.304	0.411–4.145
TNM stage (I/II *vs.* III)	<0.001	0.313	0.114–0.899	0.037	3.166	1.070–9.369
DPP10-AS1 expression (higher *vs.* lower)	0.019	2.55	1.167–5.572	0.016	2.765	1.212–6.307

OS, overall survival; HR, hazard ratio; CI, confidence interval.

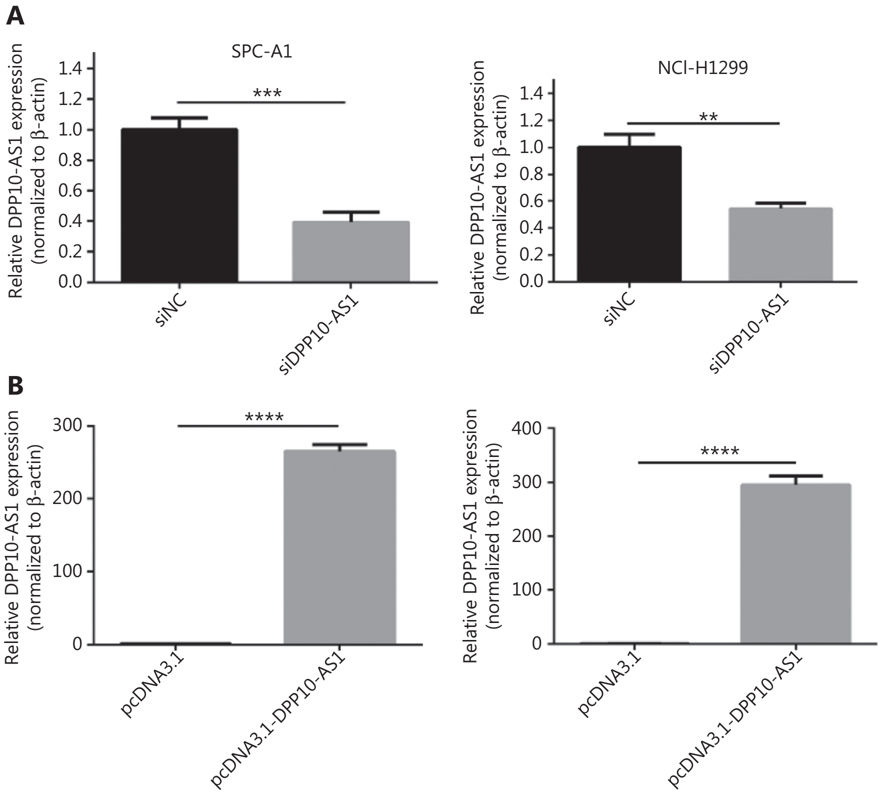

Figure S1 Knockdown and overexpression of DPP10-AS1 in lung cancer cells. (A) qRT-PCR analysis of DPP10-AS1 in SPC-A1 and NCI-H1299 lung cancer cells transfected with control siRNA (siNC) or DPP10-AS1 siRNA (siDPP10-AS1). (B) qRT-PCR analysis of DPP10-AS1 in SPC-A1 and NCI-H1299 lung cancer cells transfected with empty vector (pcDNA3.1) or DPP10-AS1 overexpression plasmid (pcDNA3.1-DPP10-AS1). Data are shown as mean ± SD from 3 independent experiments. $^{**}P < 0.01$, $^{***}P < 0.001$, $^{****}P < 0.0001$.

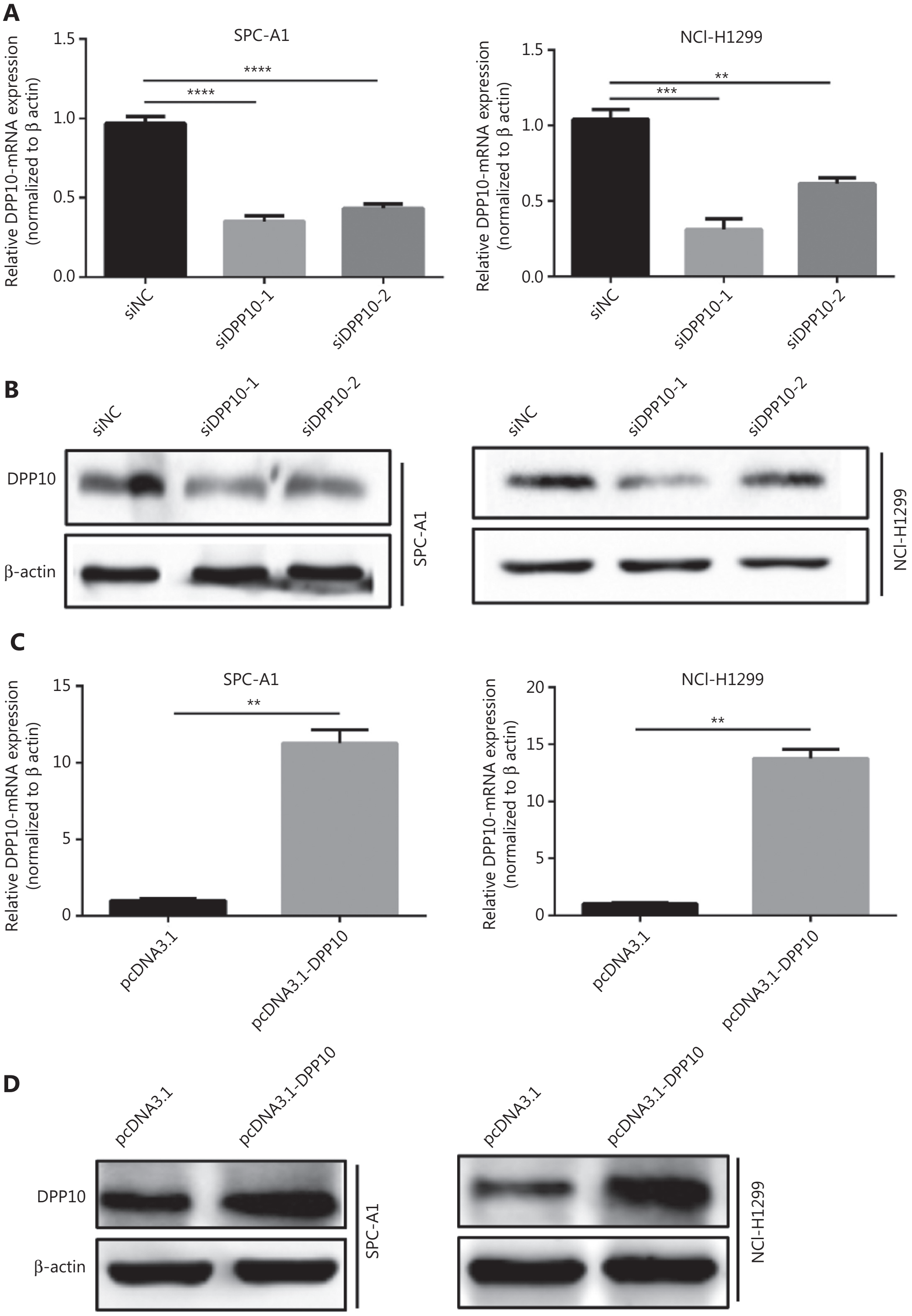

Figure S2 Knockdown and overexpression of DPP10 in lung cancer cells. (A) qRT-PCR analysis of DPP10 mRNA levels in SPC-A1 and NCI-H1299 lung cancer cells transfected with control siRNA (siNC) or DPP10 mRNA siRNA (siDPP10-1 or siDPP10-2). (B) Western blot analysis of DPP10 protein in SPC-A1 and NCI-H1299 lung cancer cells transfected with control siRNA (siNC) or DPP10 mRNA siRNA (siDPP10-1 or siDPP10-2). (C) qRT-PCR analysis of DPP10 mRNA levels in SPC-A1 and NCI-H1299 lung cancer cells transfected with empty vector (pcDNA3.1) or DPP10-AS1 overexpression plasmid (pcDNA3.1-DPP10-AS1). (D) Western blot analysis of DPP10 protein in SPC-A1 and NCI-H1299 lung cancer cells transfected with empty vector (pcDNA3.1) or DPP10-AS1 overexpression plasmid (pcDNA3.1-DPP10-AS1). Data are shown as mean ± SD from 3 independent experiments. $^{**}P < 0.01$, $^{***}P < 0.001$, $^{****}P < 0.0001$.

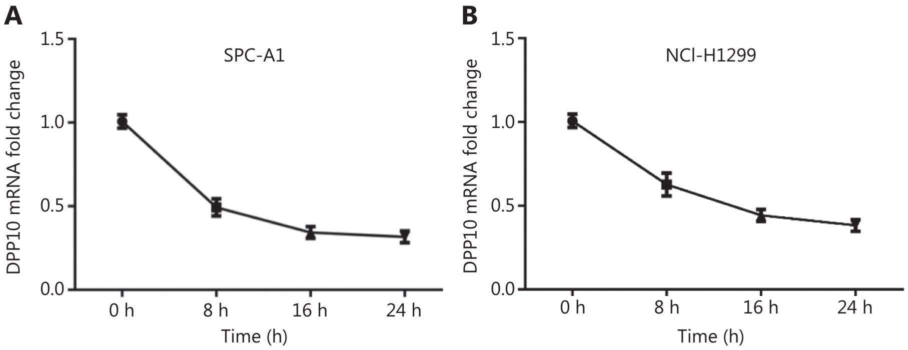

Figure S3 Effect of actinomycin D on DPP10 mRNA stability. (A) SPC-A1 cells; (B) NCI-H1299 cells.

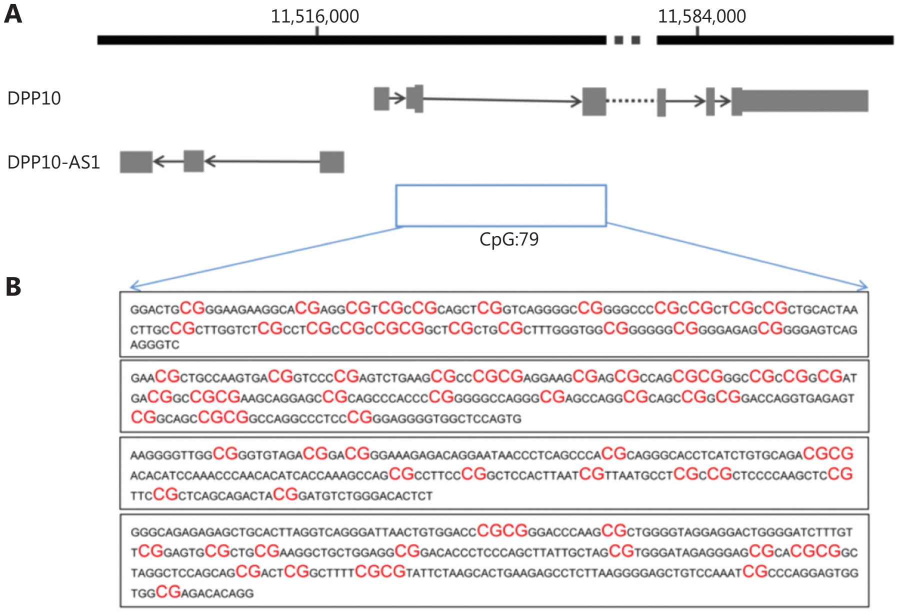

Figure S4 DNA methylation analysis of DPP10 and DPP10-AS1 genes. (A) Predicted CpG islands (gray boxes) located in 5′-flanking regions of DPP10-AS1 and DPP10 genes (black boxes). Dotted lines indicate the positions of DNA methylation analysis in this study. (B) The sites of DNA methylation (CpG sites in red) in the sequences of the DPP10 and DPP10-AS1 promoter regions.
